# Linear Mitochondrial Genome in Anthozoa (Cnidaria): A Case Study in Ceriantharia

**DOI:** 10.1038/s41598-019-42621-z

**Published:** 2019-04-15

**Authors:** Sérgio N. Stampar, Michael B. Broe, Jason Macrander, Adam M. Reitzel, Mercer R. Brugler, Marymegan Daly

**Affiliations:** 1 Departamento de Ciências Biológicas, Faculdade de Ciências e Letras, UNESP – Universidade Estadual Paulista, Assis, SP Brazil; 2 Department of Evolution, Ecology, and Organismal Biology, The Ohio State University, Columbus, OH USA; 3 Department of Biological Sciences, University of North Carolina at Charlotte, Charlotte, NC USA; 4 Department of Biology, Florida Southern College, Lakeland, FL USA; 5 Biological Sciences Department, NYC College of Technology, City University of New York, 285 Jay Street, Brooklyn, New York, 11201 USA; 6 Department of Invertebrate Zoology, American Museum of Natural History, Central Park West at 79th Street, New York, New York, 10024 USA

## Abstract

Sequences and structural attributes of mitochondrial genomes have played a critical role in the clarification of relationships among Cnidaria, a key phylum of early-diverging animals. Among the major lineages of Cnidaria, Ceriantharia (“tube anemones”) remains one of the most enigmatic in terms of its phylogenetic position. We sequenced the mitochondrial genomes of two ceriantharians to see whether the complete organellar genome would provide more support for the phylogenetic placement of Ceriantharia. For both *Isarachnanthus nocturnus* and *Pachycerianthus magnus*, the mitochondrial gene sequences could not be assembled into a single circular genome. Instead, our analyses suggest that both species have mitochondrial genomes consisting of multiple linear fragments. Linear mitogenomes are characteristic of members of Medusozoa, one of the major lineages of Cnidaria, but are unreported for Anthozoa, which includes the Ceriantharia. The inferred number of fragments and variation in gene order between species is much greater within Ceriantharia than among the lineages of Medusozoa. We identify origins of replication for each of the five putative chromosomes of the *Isarachnanthus nocturnus* mitogenome and for each of the eight putative chromosomes of the *Pachycerianthus magnus* mitogenome. At 80,923 bp, *I. nocturnus* now holds the record for the largest animal mitochondrial genome reported to date. The novelty of the mitogenomic structure in Ceriantharia highlights the distinctiveness of this lineage but, because it appears to be both unique to and diverse within Ceriantharia, it is uninformative about the phylogenetic position of Ceriantharia relative to other Anthozoa. The presence of tRNA^Met^ and tRNA^Trp^ in both ceriantharian mitogenomes supports a closer relationship between Ceriantharia and Hexacorallia than between Ceriantharia and any other cnidarian lineage, but phylogenetic analysis of the genes contained in the mitogenomes suggests that Ceriantharia is sister to a clade containing Octocorallia + Hexacorallia indicating a possible suppression of tRNA^Trp^ in Octocorallia.

## Introduction

Analyses of the mitochondrial genome have played a pivotal role in understanding relationships among Cnidaria. Foundational studies^1,2^ pointed to a clear division between Anthozoa and Medusozoa, with medusozoans sharing the derived feature of linear mitogenomes. Subsequent studies have confirmed a circular mitochondrial genome in diverse octocorals (reviewed in^3–6^ and hexacorals (reviewed by^3,7–14^ and a linear mitogenome in additional diverse medusozoans (reviewed by^3,15–17^ but see)^18,19^. Some studies have identified other characters that support a fundamental split within Cnidaria between Medusozoa and Anthozoa (e.g.^20–23^).

Comparative analyses of anthozoan mitogenomes have revealed structural genomic features like introns, transpositions, gene losses, homing endonucleases, and gene order rearrangements (e.g.^7,10,12,14,24–27,12^). This structural diversity is unexpected because anthozoan mitogenomes have some of the lowest reported rates of sequence evolution among animals (e.g.^28–31^). Within Anthozoa, the sequences and structures of the mitogenome have been used to tease apart relationships that had been controversial, such as those between scleractinians and corallimorphs^7,32^, those among Actiniaria^10^, and the relationship of zoantharians and antipatharians to other hexacorallians^8,9^.

Although mitogenomes have been more thoroughly studied in hexacorallians than in any other group of non-bilaterian metazoans^16,33^, the taxonomic sampling is highly skewed towards Actiniaria (14 of 89 mitogenomes) and Scleractinia (59 of 89)^3^ and no complete mitogenomes have been reported for any members of order/subclass Ceriantharia. Regions of the mitogenome of ceriantharians appear to evolve under different models than those of other Anthozoa^3,21,34^, suggesting that there are important differences between the mitochondrial genome of ceriantharians and those of other anthozoans.

**Figure 1:**
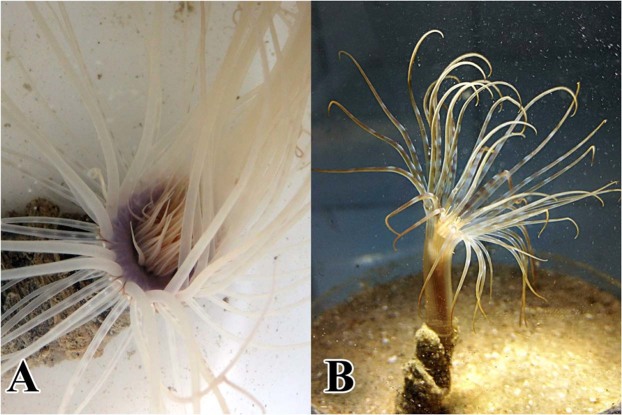
Species of Ceriantharia included in this study. (**A**) *Pachycerianthus magnus* (Nakamoto, 1919); (**B**) *Isarachnanthus nocturnus* (den Hartog, 1977).

Ceriantharia, tube anemones (Fig. 1A,B), has been an especially challenging lineage to resolve in the broader cnidarian phylogeny. Historically, they were considered sibling to the Antipatharia and grouped with them as subclass Ceriantipatharia based on similarities in the larval stage^35^. This relationship was contested based on anatomical features^36^ and later based on DNA sequence data^37^. At present, the most commonly cited relationship for Ceriantharia based on DNA sequences is as the sister to all other hexacorallians (e.g.^21,37–43^). However, Ceriantharia has also been reconstructed as the sister to Octocorallia^21^ and as the sister to all other Anthozoa^34^.

The phylogenetic position of the Ceriantharia has been difficult to test because there is little sequence data published for this group, which has the fewest sequences in GenBank of any hexacorallian order (411 sequences in nr database, 06/2018). In the phylogenomic analyses of^21^, Ceriantharia had the lowest percent recovery of genes of any anthozoan and was equally well supported in two phylogenetic positions (sister to all other Hexacorallia or sister to Octocorallia). Some of these difficulties may stem from significant differences in evolutionary rate between Ceriantharia and other Anthozoa^34^. Taxon sampling of Ceriantharia was low in the analyses of^22^ and^43^ and the group is generally represented by one or two exemplars in higher-level phylogenies (e.g.^22,23,38,41–43^). This low representation is especially significant and problematic if it is the sister lineage of a much larger group, as implied by most interpretations of its phylogeny.

### The Partial Mitogenome of *Ceriantheopsis americana*

In 2004, Sanger sequencing of the *Ceriantheopsis americana* mitogenome^44^ yielded results that suggested the presence of several linear molecules, a highly speculative and controversial conclusion at the time, and one that would remain anecdotal and unresolved until now. Using primer walking and Sanger sequencing, Brugler completely sequenced four genes (*nad1*, *cox1*, *cox2*, and *rns*) and partially sequenced two genes (*nad6* and *rnl*) and inferred their arrangement as (1) *nad1-cox1-cox2*, (2) *rns-rnl*, and (3) *nad6-rnl*. Unusual features noted at the time that led to the hypothesis that the cerianthid mitogenome was made up of several linear molecules included (1) PCRs that routinely yielded 3+ bands of different size per lane when viewed on an agarose gel (including the traditional LCO1490-HCO2198 primers used to amplify the *cox1* ‘Barcode of Life’), (2) the 3′-end of *rnl* could not be extended regardless of the cerianthid-specific primer combinations employed, (3) depending on the forward primers employed (i.e., *rnl* vs. *cox2*), two different NCRs were obtained upstream of *nad1* that both converge on the same *nad1* sequence, and (4) sequence downstream of *rns* and *nad6* converged on the same *rnl* sequence (coming from *nad6*, convergence occurred 178 bp into *rnl*; coming from *rns*, convergence occurred 124 bp into *rnl*). Two additional unique features of the *C. americana* mitogenome were that (1) *cox1* and *cox2* were fused into a single gene (2,355 bp in length) and thus continuously encoded, and (2) there was an unusually long intergenic region (1,117 bp) separating *nad6* and *rnl*. Our new data allow us to interpret and contextualize these early observations.

Recognizing the power of mitochondrial genomes to illuminate anthozoan relationships, we sequenced and characterized the mitogenome of the ceriantharians *Isarachnanthus nocturnus* and *Pachycerianthus magnus*. With the exception of preliminary sequence data from the mitogenome of *Ceriantheopsis americana*, these are the first reports of putatively complete mitochondrial genomes for members of this lineage. The genomes of *I. nocturnus* and *P. magnus* are unlike those of all other Anthozoa in being linear, but differ from one another in the arrangement of the genes and the inferred number of linear chromosomes. This surprising finding reinforces the uniqueness of Ceriantharia and underscores the difficulty in interpreting its relationship to other major groups within Cnidaria. Phylogenetic analysis based only on coding regions of these mitogenomes supports interpreting Ceriantharia as the sister to Octocorallia and Hexacorallia and thus as a third major lineage within Anthozoa. The presence of tRNA Methionine and Tryptophan in both mitogenomes of Ceriantharia recalls the situation in Hexacorallia and contrasts to the situation in Octocorallia (without tRNA^TRP^) and Medusozoa (which lack these tRNAs) and aligns with the interpretation of relationship based on genomic datasets (see more in^23^).

## Material and Methods

### Specimen sampling

The two focal species (Fig. 1) are representatives of the two orders of Ceriantharia. *Isarachnanthus nocturnus* (Hartog, 1977), order Penicillaria, was collected in São Sebastião Channel (23°49.596′S 45°25.292′W), São Paulo, Brazil (MZUSP 1478) (Ministério do Meio Ambiente - SISBIO 55566-1). *Pachycerianthus magnus*, order Spirularia, was collected from Chinwan Inner Bay (23°31.896′N 119°33.460′E), Penghu, Taiwan (MZUSP 1951) by Drs. C. Allen Chen and Hernyi Justin Hsieh. Specimens were preserved directly in 92% ethanol. Pieces of marginal tentacles were used for DNA extraction.

### Methods for obtaining and assembling mitogenomes

Libraries were prepared using an Illumina TruSeq PCR-free protocol and sequenced on an Illumina HiSeq 2500 platform yielding 250 bp paired-end reads, with an average insert size of 350 bp for *I. nocturnus* and 550 bp for *P. magnus*. The sequencing runs produced 14.2 million (m) mate-pairs for *I. nocturnus* and 15.3 m mate-pairs for *P. magnus*. The reads were evaluated for quality and adapter-contamination using FastQC^45^ and cleaned using Trimmomatic^46^ to remove adapters and low-quality regions. 12.7 m pairs were retained for *I. nocturnus* (86.1%) and 14.9 m pairs were retained for *P. magnus* (97.7%). De novo assembly was performed using DISCOVAR *de novo* v. 52488^47^ which is optimized for this type of Illumina data. The resulting assembly was converted to a BLAST database, and mitochondrial contigs identified by querying with a set of known cnidarian mitochondrial coding sequences. Trimmed reads were mapped back to the identified mitochondrial contigs using the Geneious 7.1 read mapper^48^ using High Sensitivity (Medium) default settings, and the mapped reads were reassembled de novo in Geneious to validate assembly and evaluate evenness of coverage and read-agreement. We concatenated species-specific mitochondrial contigs into a single “pseudo contig” and mapped raw reads to determine if paired end reads would map to different mitochondrial chromosomes. Regions of sequence similarity across chromosomes were identified using LASTZ v.1.02.00^49^ and GC content calculated for each chromosome^50^. The data are available via GenBank BioSample as SAMN10291198 (*Isarachnanthus nocturnus*) and SAMN10291199 (*Pachycerianthus magnus*).

Our assembly pipeline was validated by the current authors through application to other anthozoan mitochondrial genomes using identical methods of data generation. In those cases, mitochondrial genomes assembled into a single DISCOVAR contig^10^. However, in our study of these two ceriantharians, the assembly for both samples unexpectedly yielded numerous linear chromosomes. Since none of the contigs circularized and no paired-end reads bridged contigs, we attempted to extend contigs using both IMAGE^51^ with various kmer settings and the Geneious 7.1^48^ iterative read mapper using various parameter settings that balanced extensibility and accuracy. In no case did the contigs for *I. nocturnus* or *P. magnus* significantly extend: reads either falsely assembled into highly discordant, non-homologous low-complexity regions or abruptly terminated. We also independently assembled the data using NOVOPlasty v 2.5.9^52^, which is explicitly designed to assemble circular, organellar genomes. In one case, this assembler extended a single contig and circularized it; however, mapping reads back to this contig revealed that an incorporated direct repeat occurs immediately after a 3,000 bp region of minimal mapping quality, casting doubt on this assembly. We used the Phobos tandem repeat search tool^53^ but found no definitive evidence of telomeric repeats at the ends of any linear fragment. In order to check for the presence of inverted terminal repeats (ITRs) of the type found in the linear mitochondrial genomes of Medusozoans and Cubozoans we aligned each fragment to itself using LASTZ. This method successfully identifies the previously annotated ITRs in Medusozoans and Cubozoans^15^. Again, there was no evidence of telomeric structure at the ends of any assembled fragment.

Contigs were annotated using DOGMA^54^ and MITOS^55^, carefully examining MITOS scores across loci to rule out false positives, and determining ORF boundaries by transferring homologous gene annotations in Geneious from a representative selection of anthozoan and medusozoan sequences from GenBank.

### Phylogenetic mitogenome and distance analysis

**Figure 2:**
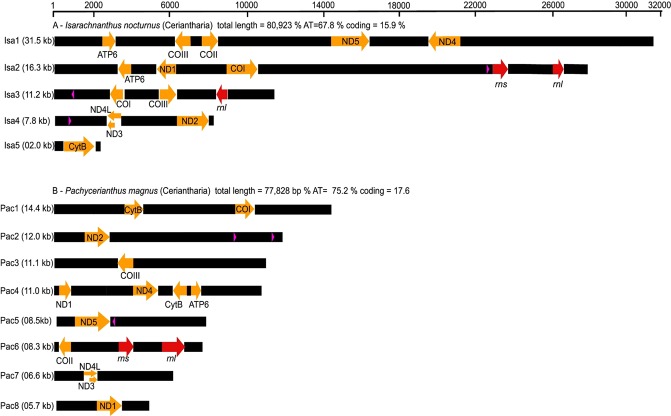
Architecture of the mitochondrial genomes of *Isarachnanthus nocturnus* (Isa1–Isa5) and *Pachycerianthus magnus* (Pac1-Pac8).

Mitochondrial genomes representing all accepted cnidarian clades – except Myxozoa – were obtained from GenBank (Table 1). Sampling aimed to increase the representation of taxonomic diversity across groups where possible. Represented taxa with complete mitogenomes from each major group were chosen to maintain equal sampling across lineages where possible. Mitochondrial genomes were partitioned by genes for the subsequent assembly of the matrix with the equivalent order of the genes. Differences in the organization of the gene sequence (Fig. 2) were not considered in the present analysis.

**Table 1:** Species included in the phylogenetic analysis.

		SPECIES	SOURCE/GENBANK CODE
CERIANTHARIA	Ceriantharia	*Isarachnanthus nocturnus*	This study - SAMN10291198
		*Pachycerianthus magnus*	This study - SAMN10291199
		*Ceriantheopsis americana*	DQ662399*, DQ662400*
HEXACORALLIA	Actiniaria	*Exaiptasia diaphana*	NC_022265
		*Alicia sansibarensis*	NC_027610
		*Antholoba achates*	KR051002
	Antipatharia	*Myriopathes japonica*	NC_027667
		*Stichopathes lutkeni*	NC_018377
	Corallimorpharia	*Corynactis californica*	NC_027102
		*Discosoma nummiforme*	NC_027100
	Scleractinia	*Dendrophyllia arbuscula*	KR824937
		*Tubastrea coccinea*	KX024566
	Zoantharia	*Palythoa heliodiscus*	NC_035579
		*Zoanthus sansibaricus*	NC_035578
OCTOCORALLIA	Alcyonacea	*Calicogorgia granulosa*	NC_023345
		*Corallium konojoi*	NC_015406
		*Dendronephthya suensoni*	NC_022809
		*Euplexaura crassa*	HQ694728
		*Muricea purpurea*	NC_029698
		*Paracorallium japonicum*	NC_015405
		*Paracorallium* sp.	AB595189
MEDUSOZOA	Cubozoa	*Alatina moseri*	KJ452776 - 83
	Hydrozoa	*Cladonema pacificum*	KT809323
		*Craspedacusta sowerbyi*	JN593332
		*Liriope tetraphylla*	KT809327
		*Pennaria disticha*	JN700950
		*Physalia physalis*	KT809328
	Scyphozoa	*Aurelia aurita*	DQ787873
		*Cassiopea andromeda*	JN700934
	Staurozoa	*Lucernaria janetae*	JN700946
		*Haliclystus antarcticus*	NC_030337

*Data not included.

The combined dataset was created for all genes from the mitochondrial genomes after alignment of individual genes with MAFFT (parameter: FFT-NS-2)^56^; this dataset was analyzed under Maximum Likelihood criteria (PhyML 3.0 and RAxML v8)^57,58^. To evaluate nodal support and to detect if support values were biased, two parametric (aLRT and aBAYES) and non-parametric (Bootstrap) bootstrap values were computed in RAxML (500 pseudoreplicates, same parameters as the original phylogenetic analysis) and additional statistical tests were performed using PhyML with SMS to infer tree model^59^. The duplicated genes (characterized by sequence similarity) were individualized and aligned in MUSCLE^60^ and compared by p-distance model in order to calculate their respective genetic distances.

### Identification of transfer RNAs

We utilized ARWEN v1.2^61^ and tRNAscan-SE v2.0^62^ to search each individual linear chromosome of *I. nocturnus* and *P. magnus* for putative tRNAs. Search parameters for ARWEN were as follows: metazoan mitochondrial tRNA genes, assuming circular topology, search wraps around ends, search both strands, use composite metazoan mitochondrial genetic code. Search parameters for tRNAscan-SE were as follows: sequence source = other mitochondrial, search mode = default (organellar), genetic code = mold/protozoan, cutoff score = 15. Only tRNAs that were located by both programs are reported.

### Identification of the origin of replication

We utilized the ‘DNA Walker’ graphing option within GraphDNA^63^ available online via the Viral Bioinformatics Resource Center [virology.uvic.ca]) to search each mitogenomic chromosome for abrupt changes in base composition bias that are characteristic of both the heavy (OriH) and light (OriL) strand origins of replication (see^64^ for usage of the DNA Walk within bamboo corals and black corals). We also searched each mitogenomic chromosome for repeats using the Tandem Repeats Finder online server (v 4.09^65^) and E-QuickTandem (v 6.5.0^66^). After locating putative origins of replication, we used the mfold web server^67^) to locate stable stem-loop configurations containing characteristic T-rich loops (see^64^ for an in-depth analysis of features typically associated origins of replication).

## Results

### The linear and fragmented mitochondrial genomes of Ceriantharia

The mitochondrial genomes we present of the ceriantharians *Pachycerianthus magnus* and *Isarachnanthus nocturnus* are the first linear and fragmented mitochondrial genomes described in Anthozoa. The obtained genomes (Fig. 2) are comprised of 77,828 bp and 80,923 bp, respectively, and are organized into eight (*P. magnus*) and five (*I. nocturnus*) contigs that likely represent chromosomes. Because we did not detect a telomere sector or something similar at the end of each set of genes, we consider these putative chromosomes, rather than definitive chromosomes.

A small percentage of the paired-end (PE) reads mapped across the possible chromosomes: 1,238 PE reads (1%) for *P. magnus* and 1,341 PE reads (0.5%) for *I. nocturnus*. These mismatches resulted in paired ends being mapped consistently to distinct positions within the chromosomes for each species (Supplemental Fig. 1). Due to the position of these mismatches, high sequence similarity across AT-rich chromosomes, and potentially duplicated chromosomal regions and associated genes, this is likely an artifact of the mapping due to their relatively low occurrence.

The ceriantharian mitochondrial genomes we have sequenced are, on average, three to four times longer than those of other cnidarians, with *I. nocturnus* having the largest animal mitochondrial genome reported to date. The size (but not content) of the mitogenome of these ceriantharians is very similar to those reported for Choanoflagellata^68^ and exceed the 21,500 bp that Brugler^44^ estimated for the mitogenome of *C. americana*. The length and organization help explain several failed attempts of Long-Range PCRs with diverse primers to obtain long mitochondrial sectors with previous mtDNA isolation (Abcam kit (AB65321); SNS & M. Maronna, pers. comm.). Previous attempts by SNS (with M. Maronna) to sequence the mitogenome based on the isolation of mitochondria, DNA extraction and subsequent sequencing on a Roche 454 GS Jr resulted in similar data for *Isarachnanthus nocturnus*, but the absence or low number of reads in some sectors meant that not all chromosome sequences could be reconstructed without breaks.

We interpret the size of ceriantharian mitogenomes as generally due to an increase in non-coding regions. Despite differences in mitogenome organization and size, the genes in the mitogenomes of *I. nocturnus* and *P. magnus* are similar in size to their homologues in other cnidarians, with the exception of ND4L (which is as much as twice the length of that in other Anthozoa) and ND6 (approximately three times the length compared to other Anthozoa). The percent of each of the ceriantharian mitogenomes that encodes proteins and RNAs was low: 19.6% in *I. nocturnus* and 20.6% in *P. magnus*. These estimates are much lower than the values obtained by Brugler^44^ for the partial mitogenome *of C. americana*, for which 6,894 bp of a total 11,225 bp (~60%) were interpreted to be coding, but the primer walking methods used in that study may have under-captured the non-coding regions and over-estimated the coding regions.

Perhaps surprising given their considerable length, we found some genes that are common in cnidarian mitogenomes are absent in these ceriantharians. In both *I. nocturnus* and *P. magnus*, we did not find the open reading frames (ORFs) polB and ORF314 (which have been reported in other anthozoans). In *P. magnus*, ATP6, CYTb, and ND1 are duplicated, with the copies differing at 34% (ATP6), 28% (CYTb), and 19% (ND1).

**Figure 3:**
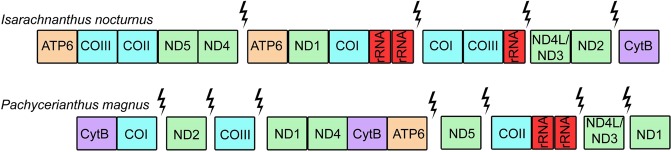
Gene order in the mitochondrial genomes of the ceriantharians *Isarachnanthus nocturnus* and *Pachycerianthus magnus*. Lightning strikes symbols indicate breaks between non-contiguous gene segments.

Thus, although the mitogenomes of *I. nocturnus* and *P. magnus* share some characteristics (linear organization and larger size) when compared to other cnidarian lineages, the organization of the genes was quite different between the two species and it was difficult to identify any conserved gene blocks between them (Fig. 3).

### Phylogenomic analysis

**Figure 4:**
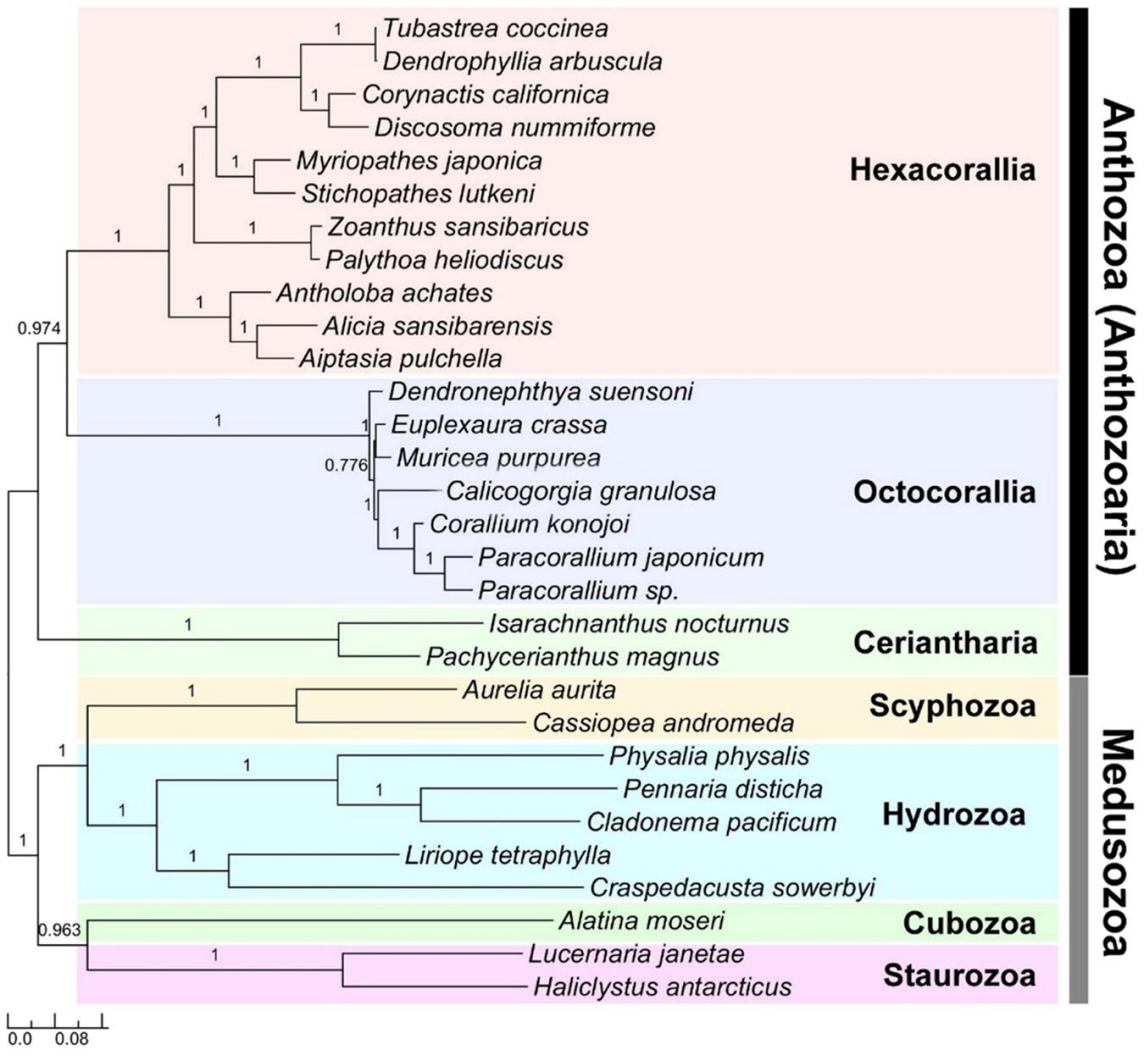
ML phylogeny of the Cnidaria based on sequences from complete mitochondrial genomes. Support values were calculated in PhyML (aBAYES).

The best tree from our maximum likelihood analysis (PHYML - model GTR, Gamma distribution parameter: 1.138, AIC = 333907.267, Log-likelihood: −291618.12823, Unconstrained likelihood: −218549.49064) of the sequences in the mitochondrial genomes of Cnidaria (Fig. 4) is similar in topology to those recovered previously (e.g.^37,69,34,23^). It includes monophyletic Medusozoa and Anthozoa, with Ceriantharia as the sister group of Hexacorallia + Octocorallia. In this tree, the monophyly of Anthozoa and of the three subgroups within it (Hexacorallia, Octocorallia, and Ceriantharia) are well supported. At the same time, the medusozoan groups have high support values despite only a small number of mitogenomes available from this group (especially in the Cubozoa and Staurozoa). The structure of the tree for Medusozoa had relatively short internal branches and relatively long terminal branches (Fig. 4).

### Transfer RNAs

**Figure 5:**
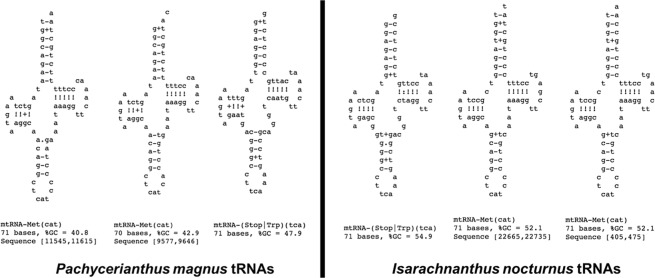
Predicted secondary structures of the three mitochondrial tRNAs: two Methionine (tRNAMet) and a single Tryptophan (tRNATrp) (ARWEN v1.2). All tRNAs are located in non-coding regions.

Within the five linear chromosomes making up the *I. nocturnus* mitogenome, we recovered three tRNAs (Fig. 5), all of which were located in non-coding regions. Transfer RNA Methionine (Met) was located on two different chromosomes (Isa2 and Isa4), while tRNA Tryptophan (Trp) was located on chromosome Isa3. Within the eight linear chromosomes making up the *P. magnus* mitogenome, we also recovered three tRNAs. Two different copies of tRNA^Met^ were located on the same chromosome (Pac2) while tRNA^Trp^ was located on chromosome Pac4.

### Origins of Replication

**Figure 6:**
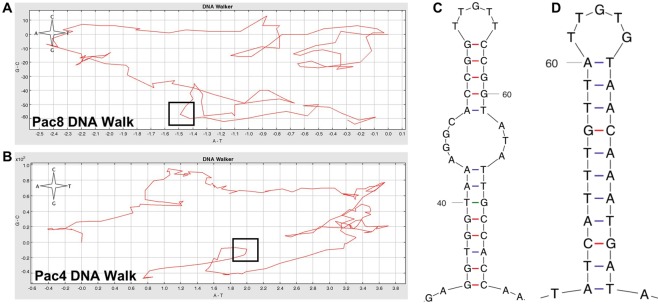
The origin of replication (Ori) was successfully located in all chromosomes. To demonstrate how we visually located the Ori, we selected the DNA Walk (**A**,**B**) and mfold (**C**,**D**) analysis of chromosome Pac8 (**A**,**C**) and Pac4 (**B**,**D**). The DNA Walk shows abrupt changes in base composition bias that are characteristic of Oris, and the mfold analysis shows stem-loop configurations containing T-rich loops that are also characteristics of Oris. For Pac8, the Ori was located at position 4,950, while for Pac4, the Ori was located at position 7,850 (both Oris are denoted by a black box). DNA Walker window size: 50.

Using a combination of DNA Walker and mfold, we successfully located the putative origin of replication in all five chromosomes of the *Isarachnanthus nocturnus* mitogenome and in all eight chromosomes of the *Pachycerianthus magnus* mitogenome. This combination of analyses searched for abrupt changes in base composition bias, stable stem-loop configurations containing T-rich loops, and repeats. While the origin of replication is often associated with inverted repeats or significant tandem repeats, in only two instances was a predicted origin of replication found associated with significant repeats (surrounding the origin of replication on chromosomes Pac5 and Pac7). Interestingly, the DNA Walks were significantly more complex than those produced to date for actiniarians, antipatharians and scleractinians (MRB personal observation). Finding a unique Ori on each individual chromosome provides definitive support that the mitogenomes of *Isarachnanthus nocturnus* and *Pachycerianthus magnus* are indeed multipartite. See Table 2 for the location of each predicted origin of replication and Fig. 6 for graphical output from DNA Walker and mfold that typically define the origin of replication.

**Table 2:** Putative location of the origin of replication (Ori) for each individual chromosome of the *Isarachnanthus nocturnus* (Isa1-5) and *Pachycerianthus magnus* (Pac1-8) mitogenome.

Chromosome	Length (bp)	Approximate Location of Ori
Isa1	31,591	17,500
Isa2	28,180	7,100
Isa3	11,231	5,900
Isa4	7,883	6,100
Isa5	2,052	250
Pac1	14,402	3,150
Pac2	12,058	4,300/6,200/10,250
Pac3	11,151	6,900
Pac4	8,469	7,850
Pac5	8,367	4,200
Pac6	6,634	5,050
Pac7	11,001	10,700
Pac8	5,746	4,950

For Pac2, there are three candidate locations for Ori, all of which are characterized by abrupt changes in base composition bias and significant stem-loop configurations; however, only the Ori at position 4,300 is also characterized by repeats.

Our analyses do not differentiate between the origin of replication on the heavy (OriH) vs. light (OriL) strand; however, based on previous analyses in closely related groups (see^63^), we hypothesize that the Oris reported here are indicative of OriH.

## Discussion

Mitochondrial genome architecture seems to be more variable among members of early-diverging clades of Metazoa than among members of the Bilateria^70,71,33^. Among non-bilaterian phyla, only Cnidaria includes major lineages for which the mitochondrial genome is not circular^1,3,33^. In Cnidaria, linear mitochondrial genomes are compelling as a synapomorphy for Medusozoa because this feature is both fairly unusual and highly consistent (reviewed by^16^). The linear mitogenomes of major medusozoan lineages are largely conserved in terms of gene order and can be related through a relatively straightforward transformation series^16^ involving fragmentation and gene re-arrangement through recombination.

A striking exception to the pattern in Cubozoa, Hydrozoa, Scyphozoa, and Staurozoa is the Myxozoa. Myxozoans are intracellular parasites with complex life cycles. Although their phylogenetic position has been difficult to assess (reviewed by^72^), they are inferred to be either highly modified medusozoans^73–75^ or the sister group (Endocnidozoa) to Medusozoa^23^. The linear mitochondrial genome characteristic among members of Medusozoa does not apply to Myxozoa, since all the myxozoan species studied to date have circular mitochondrial genomes. The mitochondrion of species in the myxozoan genus *Kudoa* are small, and the genes in them are organized into a single circular genome that is evolving more quickly than those in other Medusozoa^18^. The order of genes reported for the mitochondrial genome of *Kudoa* does not correspond to those published for other Medusozoans (cf.^16,18^). In contrast, in the myxozoan *Enteromyxum leei*, the mitogenome is organized into eight circular chromosomes^69^. This high within-lineage variation in genome architecture mirrors what we have discovered here in the Ceriantharia.

The deviation in mitogenome structure in Myxozoa does not refute the value of linear mitochondrial genomes as a synapomorphy for the Medusozoa^23^, but it does underscore that variation in mitochondrial genome structure is characteristic of Cnidaria. Likewise, the linear mitogenome of Ceriantharia we describe here should not be interpreted as proof for a particularly close relationship between Ceriantharia and Medusozoa; it is merely more evidence of plasticity in mitogenome architecture in the Cnidaria.

The present study presents evidence of the isolation of Ceriantharia in relation to Hexacorallia and Octocorallia but does not support a close relationship between Ceriantharia and Medusozoa. The gene order in the mitogenomes of Medusozoa is largely conserved among medusozoans^3,16^ and wholly different in Ceriantharia. Mitogenome architecture in anthozoan lineages is generally more variable that that of Medusozoa in structure and gene order, (see, e.g.^7–14,19,24,26,27^) other than the common occurrence of the mismatch repair gene *MutS* in the mitogenome of Octocorallia^76,77^ the nature and evolutionary importance of this variation remains unclear. The variety reported to date suggests that there may be much more diversity than has been appreciated, and a better account of this diversity may improve inference about the evolutionary transformations of the mitogenome. With respect to the phylogenetic evidence in the sequences of genes within the mitogenome, our phylogenetic reconstruction supports Ceriantharia as an isolated branch within Anthozoa, rather than a close ally of the Medusozoa (Fig. 4). This pattern of sequence affinity despite structural difference was also seen for the myxozoans *Kudoa* and *Enteromyxum*^18,78^ phylogenetic analyses of sequences place these species within or sister to the Medusozoa although the structure of their genomes is unlike those of other medusozoans.

In contrast to the conservation of gene order generally characterizing medusozoan mitochondrial genomes, we did not identify any conserved gene blocks among ceriantharians and other anthozoans. We found no consistency between our two ceriantharian species and any other published gene order from a cnidarian mitochondrial genome. The absence of any relation to the patterns observed in Octocorallia or Hexacorallia may be an indication of the phylogenetic isolation of Ceriantharia from these two groups. These differences in gene order underscore the differences in rate of gene evolution between ceriantharians and other anthozoans^34^.

The nucleotide composition of protein genes in each of the ceriantharian mitogenomes did not deviate from the general pattern seen in other Cnidaria (Table 3). In some cases (e.g. ATP6, NAD4), the nucleotide composition appears to be at an intermediate stage between Medusozoa and Anthozoa. The nucleotide composition is distinct in each of these groups and the values we found for Ceriantharia lies between them. Nevertheless, conclusive interpretations will require a greater number of species of Ceriantharia and greater sampling of Medusozoa (e.g., Cubozoa, Staurozoa).

**Table 3:** Gene properties in the mtDNA of Cnidaria after^81^.

	Ceriantharia				Anthozoa				Hydrozoa				Scyphozoa				Staurozoa				Cubozoa			
	Size	%AT	Start	End	Size	%AT	Start	End	Size	%AT	Start	End	Size	%AT	Start	End	Size	%AT	Start	End	Size	%AT	Start	End
*atp6*	702–738	66–72	A/T	A	695 ± 13	63 ± 2	ATG	A*	704 ± 1	75 ± 4	A	A*	704 ± 3	69 ± 4	A	A*	708	63 ± 1	A	A	708 ± 7	63 ± 2	AG	*
*atp8*	NP	NP	NP	NP	206 ± 22	66 ± 3	ATG	A	206 ± 3	83 ± 5	A	A*	208 ± 7	73 ± 4	AG	AG*	204	62 ± 4	A	A	210 ± 2	64 ± 4	AG	AG*
*cob*	1527–1567	65–70	ATG	A/G	1156 ± 13	64 ± 3	ATG	A/G*	1148 ± 12	73 ± 3	AG	A	1146 ± 8	66 ± 2	A	AG	1068	60 ± 2	A	?	1149	62 ± 2	A	G
*cox1*	1587–1773	58–64	AT	A	2343 ± 512	61 ± 2	ATG	A/G*	1569	67 ± 3	AG	AG	1580 ± 7	64 ± 3	A	AG	1578	61 ± 1	A	A	1569	58 ± 3	A	A
*cox2*	651–759	61–66	AT	A/G	756 ± 74	62 ± 2	ATG	A/G*	744 ± 10	73 ± 4	A	AG	746 ± 8	67 ± 4	A	AG*	747	62 ± 1	A	AG	737 ± 2	61 ± 2	A	AG*
*cox3*	690–813	59–67	AT	A	789 ± 4	61 ± 2	ATG	A/G	786	72 ± 4	A	AG	786	64 ± 3	A	AG	786	61 ± 1	A	AG	786	59 ± 3	A	AG
*nad1*	945–951	65–69	AT	A	977 ± 10	62 ± 1	ATG	A/G	989 ± 4	73 ± 4	A	AG	972 ± 5	66 ± 4	AG	A*	987	59 ± 0	A	A	987 ± 8	62 ± 3	AG	AG
*nad2*	1113–1116	66–71	AT	A	1148 ± 116	63 ± 3	ATG	A	1328 ± 32	79 ± 5	A	AG*	1323 ± 13	70 ± 5	A	AG	1346 ± 2	59 ± 4	A	A	1341	63 ± 7	A	G*
*nad3*	351–363	69–70	AT	A/G	343 ± 14	63 ± 1	ATG*	G/A	355 ± 4	77 ± 4	A	*	357 ± 6	69 ± 4	AG	A*	354	65 ± 4	A	A*	351	62 ± 4	AG	*
*nad4*	1428–1467	68–72	AT	A	1467 ± 11	63 ± 1	ATG*	G/A	1458 ± 2	76 ± 4	A	AG*	1441 ± 2	68 ± 5	A	AG*	1461	61 ± 3	A	AG*	1446	59	A	G
*nad4L*	474–675	68–71	ATG	A	298 ± 2	68 ± 1	ATG*	A	299 ± 2	79 ± 4	A	*	303 ± 1	72 ± 5	AG	A*	299 ± 2	64 ± 1	A	*	290 ± 2	67 ± 3	A	G*
*nad5*	1824–1827	68–72	ATG	A/G	1889 ± 2*	62 ± 1	ATG	AG	1832 ± 2	76 ± 4	A	AG*	1830 ± 19	68 ± 5	AG	A*	1860	60 ± 2	A	AG	1824	62 ± 1	AG	G
*nad6*	1395	70	ATA	A	582 ± 32	62 ± 3	ATG	A	556 ± 8	79 ± 5	A	AG*	564 ± 12	70 ± 5	A	AG*	553 ± 2	62 ± 2	A	A	542 ± 4	64 ± 3	A	G*
*ORF314*	NP	NP	NP	NP	?	?	?	?	291	78	A	G	313 ± 7	73 ± 8	A	A	288	62	A	A	315	64	A	A
*polB*	NP	NP	NP	NP	?	?	?	?	?	?	?	?	969	70 ± 8	A	A	1119	58	ATG	?	873	58	G	A
*rnl*	2049–2145	64–67	C	C/A	2345 ± 154	61 ± 5	NA	NA	1746 ± 9	76 ± 4	NA	NA	1818 ± 34	69 ± 5	NA	NA	1830	57 ± 1	NA	NA	769	57	NA	NA
*rns*	1126–1127	62–66	A/G	T/G	1128 ± 77	55 ± 1	NA	NA	910 ± 21	74 ± 2	NA	NA	950 ± 10	69 ± 5	NA	NA	914 ± 1	57 ± 1	NA	NA	672	62	NA	NA
*trnM*	69 ± 0	46–57	G	A/G	71 ± 0	55 ± 2	NA	NA	71 ± 1	69 ± 2	NA	NA	71 ± 0	64 ± 5	NA	NA	69 ± 0	53 ± 2	NA	NA	—	—	NA	NA
*trnW*	71 ± 0	45–52	G	G	70 ± 0	49 ± 2	NA	NA	70 ± 1	65 ± 3	NA	NA	70 ± 0	64 ± 5	NA	NA	71 ± 0	52 ± 2	NA	NA	—	—	NA	NA

*asterisk corresponds to an incomplete stop codon.

The non-coding regions in Ceriantharia are very long and account for almost 80% of the mitochondrial genome. It is in these regions that the differences between Ceriantharia and other Cnidaria are most notable. In general, non-coding regions tend to be larger in Anthozoa than in Medusozoa and represent as much as 10% of the mitochondrial genome (Octocorallia^79^). The marked increase in non-coding DNA in the mitochondrial genome of Ceriantharia is noteworthy even though the increases seem not to have been the result of a single event in the two ceriantharians studied here, because the recovered patterns are quite distinct and may indicate different events. The higher rate of mitochondrial gene evolution in Ceriantharia compared to other Anthozoa^34^ may help to explain the generation and accumulation of the non-coding regions in Ceriantharia.

Foox and colleagues^10^ posit that mitochondrial genomes may be under selective pressure to place *rns* and *rnl* in close proximity to one another. Montoya *et al*.^80^ noted that animals with adjacent rRNA genes use a “transcriptional attenuator” system to produce a nearly 50-fold excess of ribosomal RNA compared with other genes. This accelerated rate of transcription resulting from gene rearrangement may increase the productivity of a mitochondrion and, therefore, be selectively advantageous. In the *I. nocturnus* mitogenome, *rns* and *rnl* are adjacent to one another on chromosome Isa2. Additionally, *rnl* is found on three separate chromosomes (Isa1, Isa2 and Isa3), which can be explained with a similar rationale (i.e., more copies = more transcripts). In the *P. magnus* mitogenome, *rns* and *rnl* are adjacent to one another on chromosome Pac5. Evidence for increased transcription of *rns* and *rnl* is seen in the increased read coverage for those sections of the chromosomes that contain adjacent ribosomal genes (Suppl Fig. 1). These findings meet the expectations of the hypothesis that there is selective advantage to the association of *rns* and *rnl*¸ but in the absence of an ancestral gene order for the ceriantharian mitogenome or a well-resolved phylogeny for Anthozoa, we cannot determine whether these are ancestral similarities or evidence of convergent re-arrangements or speculate on the sequence of rearrangements that generated the observed gene orders.

Even though the *I. nocturnus* mitogenome is made up of five chromosomes and the *P. magnus* mitogenome consists of eight chromosomes, both mitogenomes have two tRNA^Met^ and a single tRNA^Trp^. Unlike the Octocorallia, which only have tRNA^Met^, to date, all hexacorallians have tRNA^Met^ and tRNA^Trp^. Thus, finding both tRNA^Met^ and tRNA^Trp^ in both species analyzed herein suggests that the loss of tRNA^Trp^ in octocorals is a derived condition. However, interpretation of evolutionary gain or loss of tRNAs requires a well-supported phylogeny, which remains elusive for Anthozoa. Quattrini and collegues^43^ recently developed probes to target Ultra Conserved Elements (UCEs) across a broad range of anthozoans, including the cerianthids *Cerianthus membranaceus* and *Pachycerianthus* sp. Quattrini *et al*. (*in prep*) have now sequenced 100 s of UCE across the anthozoan Tree of Life and produced the first phylogenomic tree for this group. These results, along with ongoing whole mitochondrial genome, nuclear genome and transcriptome sequencing efforts, will hopefully provide resolution as to the placement of cerianthids.

## Supplementary information

Supplementary Material

## Data Availability

The data that support the findings of this study are available from GenBank as SAMN10291198 (*Isarachnanthus nocturnus*) and SAMN10291199 (*Pachycerianthus magnus*). These will be released upon acceptance of the manuscript. Access for editors and reviewers prior to publication can be obtained through https://ftp-trace.ncbi.nlm.nih.gov/sra/review/SRP166847_20190125_103955_37d5c0b6b354bc3c790d2696b42756c9.

## References

[1] Bridge D, Cunningham CW, Schierwater B, DeSalle R, Buss LW (1992). Class-level relationships in the phylum Cnidaria: evidence from mitochondrial genome structure. Proc. Nat. Acad. Sci..

[2] Bridge D, Cunningham CW, DeSalle R, Buss LW (1995). Class-level relationships in the phylum Cnidaria: molecular and morphological evidence. Mol. Biol. Evol..

[3] Kayal E, Roure B, Philippe H, Collins AG, Lavrov DV (2013). Cnidarian phylogenetic relationships as revealed by mitogenomics. BMC Evol. Biol..

[4] Figueroa DF, Baco AR (2014). Octocoral mitochondrial genomes provide insights into the phylogenetic history of gene order rearrangements, order reversals, and cnidarian phylogenetics. Genome Biol. Evol..

[5] Wu JS, Ju YM, Hsiao ST, Hsu CH (2016). Complete mitochondrial genome of *Junceella fragilis* (Gorgonacea, Ellisellidae). Mitochondrial DNA.

[6] Poliseno A, Altuna A, Cerrano C, Wörheide G, Vargas S (2017). Historical biogeography and mitogenomics of two endemic Mediterranean gorgonians (Holaxonia, Plexauridae). Org. Div. Evol..

[7] Medina M, Collins AG, Takaoka TL, Kuehl JV, Boore JL (2006). Naked corals: skeleton loss in Scleractinia. Proc. Nat. Acad. Sci..

[8] Brugler MR, France SC (2007). The complete mitochondrial genome of the black coral *Chrysopathes formosa* (Cnidaria: Anthozoa: Antipatharia) supports classification of antipatharians within the subclass Hexacorallia. Mol. Phylogenet. Evol..

[9] Sinniger F, Chevaldonné P, Pawlowski J (2007). Mitochondrial genome of *Savalia savaglia* (Cnidaria, Hexacorallia) and early metazoan phylogeny. J. Mol. Evol..

[10] Foox J, Brugler M, Siddall ME, Rodríguez E (2016). Multiplexed pyrosequencing of nine sea anemone (Cnidaria: Anthozoa: Hexacorallia: Actiniaria) mitochondrial genomes. Mitochondrial DNA Part A..

[11] Shi X (2016). Complete mitochondrial genome of disc coral *Turbinaria peltata* (Scleractinia, Dendrophylliidae). *Mitochondrial*. DNA.

[12] Chi SI, Johansen SD (2017). Zoantharian mitochondrial genomes contain unique complex group I introns and highly conserved intergenic regions. Gene.

[13] Zhang L, Zhu. Q (2017). Complete mitochondrial genome of the sea anemone, *Anthopleura midori* (Actiniaria: Actiniidae). Mitochondrial DNA.

[14] Zhang B, Zhang YH, Wang X, Zhang HX, Lin Q (2017). The mitochondrial genome of a sea anemone *Bolocera* sp. exhibits novel genetic structures potentially involved in adaptation to the deep‐sea environment. Ecol. Evol..

[15] Smith DR (2012). First complete mitochondrial genome sequence from a box jellyfish reveals a highly fragmented linear architecture and insights into telomere evolution. Genome Biol Evol..

[16] Kayal E (2015). Phylogenetic analysis of higher-level relationships within Hydroidolina (Cnidaria: Hydrozoa) using mitochondrial genome data and insight into their mitochondrial transcription. PeerJ.

[17] Li HH, Sung PJ, Ho HC (2016). The complete mitochondrial genome of the Antarctic stalked jellyfish, *Haliclystus antarcticus* Pfeffer, 1889 (Staurozoa: Stauromedusae). *Genome*. Data.

[18] Takeuchi F (2015). The mitochondrial genomes of a myxozoan genus *Kudoa* are extremely divergent in Metazoa. PLoS One.

[19] Dubin, A., Chi, S. I., Emblem, Å. Moum, T. & Johansen, S. D. Deep-water sea anemone with a two-chromosome mitochondrial genome. *Gene* **692**, 195-200 (2019). 10.1016/j.gene.2018.12.074 30641219

[20] Marques, A. C. & Collins, A. G. Cladistic analysis of Medusozoa and cnidarian evolution. *Invert Biol*. **123**, 23-42 (2004).

[21] Daly. M (2007). The phylum Cnidaria: A review of phylogenetic patterns and diversity 300 years after Linnaeus. Zootaxa.

[22] Zapata F (2015). Phylogenomic analyses support traditional relationships within Cnidaria. PLoS One.

[23] Kayal E (2018). Phylogenomics provides a robust topology of the major cnidarian lineages and insights on the origins of key organismal traits. BMC Evol Biol..

[24] Beagley CT, Okimoto R, Wolstenholme DR (1998). The mitochondrial genome of the sea anemone *Metridium senile* (Cnidaria): introns, a paucity of tRNA genes, and a near-standard genetic code. Genetics.

[25] Fukami H, Chen CA, Chiou CY, Knowlton N (2007). Novel group I introns encoding a putative homing endonuclease in the mitochondrial cox1 gene of scleractinian corals. J. Mol. Evol..

[26] Emblem Å (2014). Sea anemones possess dynamic mitogenome structures. Mol. Phylogenet. Evol..

[27] Emblem Å, Karlsen BO, Evertsen J, Johansen SD (2011). Mitogenome rearrangement in the cold-water scleractinian coral *Lophelia pertusa* (Cnidaria, Anthozoa) involves a long-term evolving group I intron. Mol Phylogenet Evol.

[28] Shearer TL, Van Oppen MJH, Romano SL, Wörheide G (2002). Slow mitochondrial DNA sequence evolution in the Anthozoa (Cnidaria). Mol Ecol..

[29] Huang D, Meier R, Todd PA, Chou LM (2008). Slow mitochondrial COI sequence evolution at the base of the metazoan tree and its implications for DNA barcoding. J. Mol. Evol.

[30] Chen IP (2009). Comparative analyses of coding and noncoding DNA regions indicate that *Acropora* (Anthozoa: Scleractina) possesses a similar evolutionary tempo of nuclear vs. mitochondrial genomes as in plants. Mar Biotechnol..

[31] Daly M, Gusmão LC, Reft AJ, Rodríguez E (2010). Phylogenetic signal in mitochondrial and nuclear markers in sea anemones (Cnidaria, Actiniaria). Integ. Comp. Biol..

[32] Kitahara MV (2014). The “Naked Coral” hypothesis revisited – evidence for and against scleractinian monophyly. PLoS One.

[33] Lavrov DV, Pett W (2016). Animal mitochondrial DNA as we do not know it: mt-genome organization and evolution in Nonbilaterian lineages. Genome Biol Evol..

[34] Stampar SN, Maronna MM, Kitahara MV, Reimer JD, Morandini AC (2014). Fast-evolving mitochondrial DNA in Ceriantharia: A reflection of Hexacorallia paraphyly?. PLoS One.

[35] van Beneden ELesAnthozoaires (1897). de la “Plankton-Expedition”. Ergebn Plankton-Exp Humbolt-Stiftung..

[36] Schmidt, H. On the evolution in the Anthozoa in *Proceedings of the 2nd international symposium on coral reefs* (ed. Cameron, A. M *et al*.) 533–560 (The Great Barrier Reef Committee, 1974).

[37] Chen CA (1995). Systematic relationships within the Anthozoa (Cnidaria: Anthozoa) using the 5′-end of the 28S rDNA. Mol. Phylogenet. Evol..

[38] France SC, Rosel PE, Agenbroad JE, Mullineaux LS, Kocher TD (1996). DNA sequence variation of mitochondrial large-subunit rRNA provides support for a two-subclass organization of the Anthozoa (Cnidaria). Mol Mar Biol Biotech..

[39] Berntson EA, France SC, Mullineaux LS (1999). Phylogenetic relationships within the class Anthozoa (phylum Cnidaria) based on nuclear 18S rDNA sequences. Mol. Phylog. Evol..

[40] Won J, Rho B, Song J (2001). A phylogenetic study of the Anthozoa (phylum Cnidaria) based on morphological and molecular characters. Coral Reefs.

[41] Daly M, Fautin DG, Cappola VA (2003). Systematics of the Hexacorallia (Cnidaria: Anthozoa). Zool J Linn Soc..

[42] Rodríguez E (2014). Hidden among sea anemones: the first comprehensive phylogenetic reconstruction of the order Actiniaria (Cnidaria, Anthozoa, Hexacorallia) reveals a novel group of hexacorals. PLoS One.

[43] Quattrini AM (2018). Universal target‐enrichment baits for anthozoan (Cnidaria) phylogenomics: New approaches to long‐standing problems. Mol. Ecol. Res..

[44] Brugler, M. R. The complete mitochondrial DNA sequence of the black coral *Chrysopathes formosa* (Antipatharia) and six non-contiguous mitochondrial genes of the tube anemone *Ceriantheopsis americanus* (Ceriantharia): implications for cnidarian phylogeny. *Doctoral dissertation, Graduate School of the College of Charleston*. 2004.

[45] Andrews, S. FastQC: a quality control tool for high throughput sequence data. Version 1 [FastQC]. Available from, http://www.bioinformatics.babraham.ac.uk/projects/fastqc. 2018 Jun 18.

[46] Bolger AM, Lohse M, Usadel B (2014). Trimmomatic: a flexible trimmer for Illumina sequence data. Bioinformatics.

[47] Weisenfeld NI (2014). Comprehensive variation discovery in single human genomes. Nature Genetics.

[48] Kearse M (2012). Geneious Basic: an integrated and extendable desktop software platform for the organization and analysis of sequence data. Bioinformatics.

[49] Harris, R. S. Improved pairwise alignment of genomic DNA. PhD thesis. Penn State University, Computer Science and Engineering, 2007.

[50] Richard, D. GC Content in sliding window - GitHub repository. Available from, https://github.com/DamienFr/GC-content-in-sliding-window- 2018 Jun 18.

[51] Tsai IJ, Otto TD, Berriman M (2010). Improving draft assemblies by iterative mapping and assembly of short reads to eliminate gaps. Genome Biol..

[52] Dierckxsens N, Mardulyn P, Smits G (2016). NOVOPlasty: de novo assembly of organelle genomes from whole genome data. Nucleic Acids Res..

[53] Mayer, C. Phobos 3.3.11. Available from, http://www.rub.de/spezzoo/cm/cm_phobos.htm 2018 Jun 18.

[54] Wyman SK, Jansen RK, Boore JL (2004). Automatic annotation of organellar genomes with DOGMA. Bioinformatics.

[55] Bernt M (2013). MITOS: improved de novo metazoan mitochondrial genome annotation. Mol. Phylog. Evol..

[56] Katoh, K., Rozewicki, J. & Yamada, K. D. MAFFT online service: multiple sequence alignment, interactive sequence choice and visualization. *Brief. Bioinform*. 2017 Sep 6. 10.1093/bib/bbx108 PMC6781576 28968734

[57] Guindon S (2010). New algorithms and methods to estimate maximum-likelihood phylogenies: assessing the performance of PhyML 3.0. Syst Biol..

[58] Stamatakis A (2014). RAxML version 8: a tool for phylogenetic analysis and post-analysis of large phylogenies. Bioinformatics.

[59] Lefort V, Longueville JE, Gascuel O (2017). SMS: smart model selection in PhyML. Mol Biol Evol..

[60] Edgar RC (2004). MUSCLE: multiple sequence alignment with high accuracy and high throughput. Nucl. Acids Res..

[61] Laslett D, Canbäck B (2007). ARWEN: a program to detect tRNA genes in metazoan mitochondrial nucleotide sequences. Bioinformatics.

[62] Lowe TM, Chan PP (2016). tRNAscan-SE On-line: integrating search and context for analysis of transfer RNA genes. Nucleic Acids Res..

[63] Thomas JM, Horspool D, Brown G, Tcherepanov V, Upton C (2007). GraphDNA: a Java program for graphical display of DNA composition analyses. Bioinformatics.

[64] Brugler MR, France SC (2008). The mitochondrial genome of a deep-sea bamboo coral (Cnidaria, Anthozoa, Octocorallia, Isididae): genome structure and putative origins of replication are not conserved among octocorals. J. Mol. Evol..

[65] Benson G (1999). Tandem repeats finder: a program to analyze DNA sequences. Nucleic Acids Res..

[66] Rice P, Longden I, Bleasby A (2000). EMBOSS: the European molecular biology open software suite. *Trends*. Genetics.

[67] Zuker M (2003). Mfold web server for nucleic acid folding and hybridization prediction. Nucleic Acids Res..

[68] Burger G, Forget L, Zhu Y, Gray MW, Lang BF (2003). Unique mitochondrial genome architecture in unicellular relatives of animals. Proc. Nat. Acad. Sci.

[69] Song JI, Won JH (1997). Systematic relationship of the anthozoan orders based on the partial nuclear 18S rDNA sequences. Korean J Biol Sci..

[70] Lavrov DV (2012). Mitochondrial DNA of *Clathrina clathrus* (Calcarea, Calcinea): six linear chromosomes, fragmented rRNAs, tRNA editing, and a novel genetic code. Mol Biol Evol..

[71] Osigus HJ, Eitel M, Bernt M, Donath A, Schierwater B (2013). Mitogenomics at the base of Metazoa. Mol Phylogen Evol..

[72] Foox J, Siddall ME (2015). The road to Cnidaria: history of phylogeny of the Myxozoa. J Parasitol..

[73] Evans NM, Lindner A, Raikova EV, Collins AG, Cartwright P (2008). Phylogenetic placement of the enigmatic parasite, *Polypodium hydriforme*, within the phylum Cnidaria. BMC Evol Biol..

[74] Evans NM, Holder MT, Barbeitos MS, Okamura B, Cartwright P (2010). The phylogenetic position of Myxozoa: exploring conflicting signals in phylogenomic and ribosomal data sets. Mol Biol Evol..

[75] Chang ES (2015). Genomic insights into the evolutionary origin of Myxozoa within Cnidaria. Proc. Nat. Acad. Sci..

[76] Pont-Kingdon GA (1995). A coral mitochondrial mutS gene. Nature.

[77] Bilewitch JP, Degnan SM (2011). A unique horizontal gene transfer event has provided the octocoral mitochondrial genome with an active mismatch repair gene that has potential for an unusual self-contained function. BMC Evol. Biol..

[78] Yahalomi D (2017). The multipartite mitochondrial genome of *Enteromyxum leei* (Myxozoa): eight fast-evolving megacircles. Mol. Biol. Evol..

[79] Park E, Song JI, Won YJ (2011). The complete mitochondrial genome of *Calicogorgia granulosa* (Anthozoa: Octocorallia): potential gene novelty in unidentified ORFs formed by repeat expansion and segmental duplication. Gene.

[80] Montoya J, Gaines GL, Attardi G (1983). The pattern of transcription of the human mitochondrial rRNA genes reveals two overlapping transcription units. Cell.

[81] Kayal E (2011). Evolution of linear mitochondrial genomes in medusozoan cnidarians. Genome Biol. Evol..

