# Supplementary material for: Linear Mitochondrial Genome in Anthozoa (Cnidaria): A Case Study in Ceriantharia
Source: Sci Rep. 2019 Apr 15;9:6094. doi: 10.1038/s41598-019-42621-z (PMC6465557; doi:10.1038/s41598-019-42621-z)
Supplement: Supplementary file 1 — Supplementary Material [file 41598_2019_42621_MOESM1_ESM.pdf]

Linear Mitochondrial Genome in Anthozoa (Cnidaria): A Case Study in Ceriantharia

Sérgio N. Stampar, Michael B. Broe, Jason Macrander, Adam M. Reitzel, Mercer R. Brugler and Marymegan Daly

Supplementary Material – Figure 1

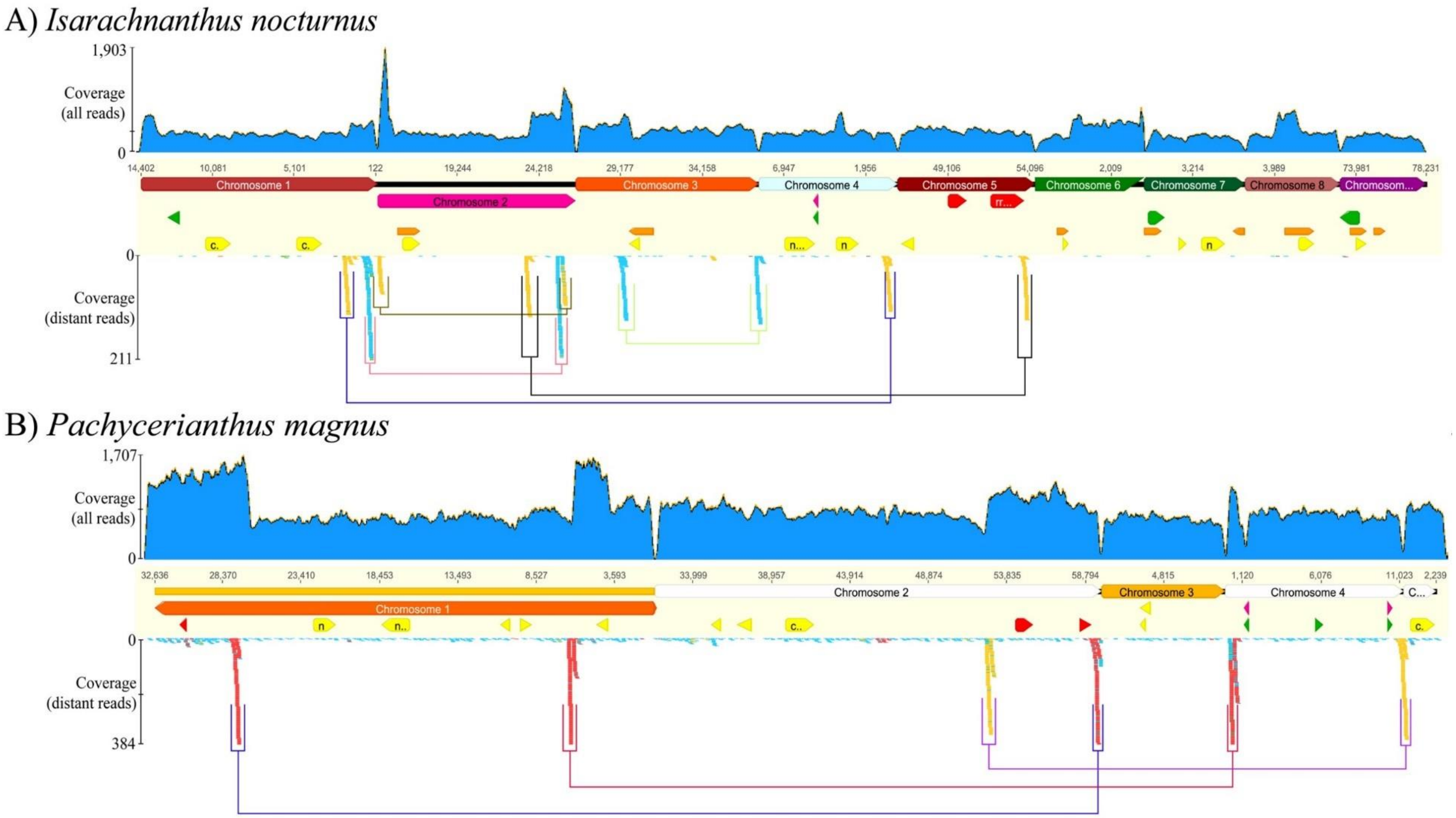

Suppl. Figure 1 - Mapped reads for each mitochondrial genome described in this study.
